# The mechanisms, diagnosis and management of mitral regurgitation in mitral valve prolapse and hypertrophic cardiomyopathy

**DOI:** 10.15190/d.2016.8

**Published:** 2016-06-30

**Authors:** Mihaela Octavia Popa, Ana Maria Irimia, Mihai Nicolae Papagheorghe, Elena Miruna Vasile, Simona Andreea Tircol, Raluca Andreea Negulescu, Catalina Toader, Robert Adam, Lucian Dorobantu, Cristina Caldararu, Maria Alexandrescu, Sebastian Onciul

**Affiliations:** Carol Davila University of Medicine and Pharmacy, Bucharest, Romania; Department of Cardiovascular Surgery, Monza Hospital, Bucharest, Romania; Department of Cardiology, Monza Hospital, Bucharest, Romania; Department of Radiology and Imaging Sciences, Monza Hospital, Bucharest, Romania; Department of Cardiology, Floreasca Clinical Emergency Hospital, Bucharest, Romania

**Keywords:** Mitral valve prolapse, hypertrophic cardiomyopathy, leaflet elongation, left ventricular outflow obstruction, systolic anterior motion, echographic evaluation, mitral valve repair, septal myectomy and mitral valve repair

## Abstract

Valvular disease is a frequent cardiac pathology leading to heart failure and, ultimately, death. Mitral regurgitation, defined as the inability of the two mitral leaflets to coapt, is a common valvular disease and a self sustained pathology. A better understanding of the mitral valve histological layers provides a better understanding of the leaflet and chordae changes in mitral valve prolapse. Mitral valve prolapse may occur in myxomatous degenerative abnormalities, connective tissue disorders or in sporadic isolated cases. It is the most common mitral abnormality of non-ischemic cause leading to severe surgery-requiring mitral regurgitation. In addition to standard echocardiographic investigations, newly implemented three-dimensional techniques are being used and they permit a better visualisation, from the so-called ‘surgical view’, and an improved evaluation of the mitral valve. Hypertrophic cardiomyopathy is the most frequent inherited myocardial disease caused by mutations in various genes encoding proteins of the cardiac sarcomere, leading to a marked left ventricular hypertrophy unexplained by other comorbidities. The pathological echocardiographic hallmarks of hypertrophic cardiomyopathy are left ventricular hypertrophy, left ventricular outflow tract obstruction and systolic anterior motion of the mitral valve. The systolic anterior motion of the mitral valve contributes to the development of mitral regurgitation and further narrows the left ventricular outflow tract, leading to more severe symptomatology. Cardiac magnetic resonance imaging accurately measures the left ventricular mass, the degree of diastolic function and it may also be used to distinguish phenotypic variants. The clinical outcome of patients with these pathologies is mostly determined by the selected option of treatment. The purpose of surgical correction regarding mitral valve involvement is to restore valvular competence. Surgery has proven to be the only useful treatment in preventing heart failure, improving symptomatology and reducing mortality. Our approach wishes to enhance the understanding of the mitral valve’s involvement in hypertrophic cardiomyopathy and mitral valve prolapse from genetic, haemodynamic and clinical perspectives, as well as to present novelties in the grand field of treatment.

## SUMMARY

1. *Introduction*
2. *Mitral valve structure and haemodynamics*
3. *Genetic causes*
4. *Leaflet elongation and thickening*
5. *Diagnosis*
  - * 5.1 Mitral valve prolapse*
  - * 5.2 Mitral valve in HCM*
6. *Treatment*
  - *6.1 Mitral Valve Prolapse Management*
  - *6.2 Management of mitral valve in HCM*
  - *6.3 Clinical outcome*
  - *6.4 Future perspectives *
7. *Conclusions*

## 1. Introduction

Valvular disease is a frequent cardiac pathology worldwide, which ultimately may lead to heart failure. The incidence of valvular disease in industrialized countries is increasing, as a result of the increase in general life expectancy.^1^The prevalence of valvular heart disease was reported to be 2.5% in 2015, in the USA alone^1^, with no difference in frequency between genders.^2^ The prevalence rises with age, affecting mostly people older than 65 years of age. The most frequent entities are aortic stenosis and mitral regurgitation (MR).^3^

MR is defined as the inability of the two mitral leaflets to coapt, leading to a systolic retrograde blood flow, from the left ventricle (LV) to the left atrium, which will result in volume overload of the left heart chambers and dilation of the mitral annulus. As a self-sustained pathology, it usually aggravates over time, leading to left heart failure.^1^ Other complications include arrhythmias such as atrial fibrillation (AF), endocarditis and death.^4^ The causes that lead to MR are classified as ischemic (due to the coronary insufficiency) and nonischemic. MR can also be classified as functional, due to annular dilation (as seen in dilated cardiomyopathies) or organic, in pathologies that affect the intrinsic structure of the valve, as seen in mitral valve prolapse (MVP).^1^ MR may also arise due to a congenital or an acquired pathological state.^5^

Until now, the mitral valve (MV) was considered a passive structure. It has recently been demonstrated that it can react to the morphological changes that involve the LV and that it may also alter its structure irrespective of the state of the nearby structures.^5,6^ However, concurrent with ventricular remodeling, mitral leaflets can become inadequately small (as seen in dilated cardiomyopathies) or suffer a process of elongation. In hypertrophic cardiomyopathy (HCM), the leaflet elongation exceeds the needs of the reduced volume of the LV, further aggravating the left ventricular outflow tract (LVOT) obstruction and can predispose to systolic anterior motion (SAM) of the MV.^5,7^ In MVP, the leaflets are long and exhibit structural fibroelastic abnormalities.^5,8^

The surgical treatment has an important role in the natural history of the disease and it has been proven to be the only treatment that may efficiently prevent heart failure, improving symptoms and reducing mortality by 70%. The best results were seen in asymptomatic MR, which supports the importance of an early and accurate diagnosis.^1^

This review highlights the mechanisms, the diagnosis and the current and future management of patients with mitral regurgitation, in the context of mitral valve prolapse and/or hypertrophic cardiomyopathy.

## 2. Mitral valve structure and haemodynamics

The mitral apparatus is composed of two leaflets, the anterior and the posterior one respectively, the mitral annulus, and the subvalvular complex, which is composed by the papillary muscles and the chordae tendineae. Each of these structures has an important role in maintaining the function of the valve.^9^ The anterior leaflet is relatively fixed and its insertion occupies 1/3 of the circumference of the annulus, while the posterior one occupies the rest of the 2/3 and is more flexible, being the most susceptible to prolapse.^9,10^ Each of the leaflets is divided by two notches in three parts, called scallops, three anterior (A1, A2, A3) and three posterior (P1, P2, P3). This division is more evident in the posterior leaflet, and is an important aspect in the diagnosis and surgical treatment of MVP.^10,11^ The two leaflets are attached by their bases to the mitral annulus, whereas the edge and midportion of the leaflets are attached to the papillary muscles by means of the chordae tendineae. This system maintains an unidirectional blood flow during diastole, from the atrium to the ventricle and closes completely, preventing retrograde flow from the ventricle to the atrium, during systole.^1,5^

Histologically, it has been shown that the leaflets comprise, from the annulus to the edge, smooth muscle fibers, nerve fibers^5,12^, dense collagen fibers, fibroblasts and glycosaminoglycans that easily absorb water and disperse mechanical stress when the two leaflets coapt.^5,13^ The chordae tendineae are strong structures with viscoelastic properties, which are composed of a core of collagen and a sheet of elastin.^5^ Due to the presence of the atrial smooth muscle fibers both on the annulus and the basal portion of the leaflet, the annulus will contract during presystole, further helping coaptation of the leaflets. It has been shown that the contraction of the annulus can be activated through ventricular smooth muscle fibers as well, although in this case MR may occur.^14^

In transverse sections, the valve is composed of three layers – *atrialis *(composed of collagen, elastin, atrial smooth muscle fibers and nerve endings), *spongiosa* (glycosaminoglycans, increasing in number and varying in structure towards the edge of the leaflet) and *ventricularis *(composed mostly of collagenous thick fibers, attached to the annulus).^4,5^ These components are modified in diseases such as MVP.^15^ On both sides, the leaflets are covered with a thin endothelial layer.^4^ Moreover, in the structure of the valve, there are valvular interstitial cells (VICs) that are connected through signaling pathways with the cells which compose the endothelial layers. When valve homeostasis is disrupted, myofibroblastic transition of the interstitial cells occurs, therefore promoting valve thickening.^4,5,16^

The relative proportion of the valvular histological components varies with age, over time decreasing in cellularity and glycosaminoglycans, while increasing in fragmentation of the elastin network, collagen fibers and calcifications, thus promoting stiffness and loss of function of the valve.^17,18^

## 3. Genetic causes

*Mitral Valve Prolapse*

MVP may occur in myxomatous degenerative abnormalities such as Barlow’s disease, connective tissue disorders such as Marfan’s Syndrome (MFS) and it may also be found in sporadic isolated cases.^19^

Through chromosomal mapping, it has been shown that myxomatous MVP has a genetic heterogeneity which implies mutations located on chromosomes 16, 11 and 13.^20-22^ Autosomal dominant and X-linked forms have also been cited.^19^

Mutations of the fibrillin-1 (FBN1) gene which encodes the fibrillin-1 protein and mutations of the transforming growth factor β (TGF-β) receptor 2 gene are responsible for the occurrence of Marfan’s syndrome, as well as MVP.^23,24^ Fibrillin-1 protein is the principal component of extracellular matrix (ECM) microfibrils and also one of the regulating factors of TGF-β activity, by inhibiting it.^24^ In patients with fibrillin deficiency, an enhanced activity of TGF-β was noticed. Also, in patients with TGF-β receptor 2 mutations, the altered receptor presented increased activity for its agonist.^23,24^

It has also been shown that cells from valves with FBN1 mutations presented decreased apoptosis rates and increased cell proliferation.^24^

Another gene implicated in the etiopathogenesis of MVP is the Filamin A gene. An association between the up-regulation of Filamin A gene and X-linked forms of myxomatous valvulopathies has been shown.^24,25^ Filamin-A is an actin-binding protein, involved in maintaining membrane stability and sheltering cells against injury and also has the function to positively regulate the expression of TGF-β.^24^

An up-regulation of TGF-β – inducible gene H3, has also been found in myxomatous degenerated valves, which supports the theory that increased levels of this factor contribute to the cause of MVP. Moreover, high levels of TGF-β have also been found in the mitral valves of patients with isolated myxomatous MVP.^24^

Another potential gene incriminated as one of the causes of MVP is the Dachsous Cadherin-Related 1 (DCHS1) gene. There is evidence that down-regulation of this gene determines abnormal valvular development, with structural valvular changes, as seen in MVP, with family aggregation and mostly involvement of the posterior leaflet.^26^

*Hypertrophic Cardiomyopathy*

HCM is the most frequent inherited cardiomyopathy^27^ characterized by high grade left ventricular hypertrophy (LVH) which cannot be explained by other comorbidities (such as heightened afterload or restrictive diseases).^28,29^

HCM is an autosomal dominant disease^30^, caused by mutations of the proteins that make up the sarcomere, in contrast to dilated cardiomiopathies, where mutations of cytoskeletal proteins are incriminated.

Until recently, it has been considered that mutations in 8 genes that codify the sarcomeric proteins are responsible for HCM, but it has been proven that up to 43 altered genes may modify the structure of myocites, further being involved in the etiology of this disease.^30^ The most frequently occurring mutations associated with HCM are found in cardiac myosin-binding protein C 3 (MYBPC3) gene, cardiac muscle β-myosin heavy chain 7 (MYH7) gene and troponin T protein type 2 (TNNT2) gene.^30-32^ Mutations of genes encoding cytoskeletal proteins may also be involved in the etiology of HCM.^27^ All of the above support the idea of a heterogenic etiology and phenotypic appearance of HCM.

These mutations can interact at three main levels: sarcomeric function, metabolic pathways and calcium signaling pathways.^30^ It has been shown that some mutations predispose to an enhanced calcium sensitivity, which can lead to metabolic impairment and cytotoxicity.^27^ Heightened oxidative stress and altered expression levels of SERCA and phospholamban have also been cited in the pathology of HCM.^33,34^ Also it has been shown that mutations in the cardiac ryanodine receptor (mainly implicated in the genesis of arrhythmias) may be correlated with HCM, mostly with symmetric apical forms.^30^

In up to 5% of cases HCM can be accompanied by skeletal muscle disorders, such as neuromuscular diseases.^27,30^ Sporadic cases of HCM may also appear and are explained by a novel mutation or incomplete gene penetrance in the patient’s parents.^30^

## 4. Leaflet elongation and thickening

*Mitral valve prolapse*

MVP presents two main phenotypes that are recognized in the literature: myxomatous degeneration – Barlow’s disease and fibroelastic dysplasia.^35,36^ Both of these degenerative processes are associated with leaflet elongation and thickening, as well as chordal abnormalities.^8,37^

Heart valve development may help understand signaling pathways involved in MVP. Primordial valves derive from the cardiac cushions which are regional swellings of the ECM.^38^ Members of the TGF-β family are implicated in mediating signals in the cardiac cushion. These reciprocal signals between the endocardial and myocardial cell layers promote the transformation of valvular endothelial cells (VECs) into (VICs).^39^ It has been shown, both on animal models^40^ and in human in vitro studies that TGF-β up-regulation plays an essential role in the pathogenesis of MVP. TGF-β activates VICs towards a myofibroblast-like phenotype and causes excessive remodeling of the ECM.^41,42^

In myxomatous degeneration, the disruption of the ECM homeostasis is an essential mediator of the pathological changes.^38,43^ VICs modify their phenotype to myofibroblast-like cells characterised by the expression of vimentin and α-smooth muscle actin and lack of expression of myosin heavy chain isoforms 1 or 2- markers of differentiated smooth muscle cells.^44,45^ Normal VICs are responsible for maintaining a tight balance between degradation and synthesis of the ECM proteins by means of enzymes such as matrix metalloproteinases (MMPs) - collagenase and gelatinases, as well as tissue inhibitors of MMPs.^8,37^ In contrast, modified VICs are responsible for an exceedingly high rate of collagen and elastin degradation compared to the production rate due to increased concentrations of various proteolytic enzymes.^45-47^ Furthermore, possible fibrocytes capable of differentiating into myofibroblasts that both produce and degrade collagen and elastin, may be present in myxomatous valves.^38^

The excessive degradation of the ECM results in an extracellular accumulation of mucopolysaccharides which leads to the augmentation of the *spongiosa*.^43,48^ These muccoplysaccharides overflow onto *ventricularis* collagenous fibers and *atrialis* elastic fibers resulting in reduction of the collagen fibers and altered architecture of the leaflet. Nevertheless, there is an increase in type III collagen to the detriment of type I which is more resistant to traction.^43,48^ These changes promote valve thickening. The chordae undergo changes which reside in mucoid infiltration with glycosaminoglycans leading to the disrupture of their fibrous core.^43,49^ Grande-Allen et al. concluded that the predominant glycosamino-glycans in which the chordae are abundant are chondroitin/dermatan 6-sulfate, in contrast to leaflets which contained predominantly hyaluronan.^49^ These findings may account for both chordal and leaflet reduced tensile strength.^50^

There is evidence showing that non-ruptured choardae tendinae may be hidden (be covered up) by superimposed fibrous tissue of the leaflets, thus partaking to their thickening. Nevertheless, ruptured chordae may be partially included in the posterior superimposed fibrous tissue of the leaflets.^51^ The posterior superimposed fibrous tissue has been proven to be the main cause of posterior leaflet thickening rather than the expansion of the *spongiosa*. Furthermore, it is thought to be the result of the abnormal contact between leaflets and chordae.^52^

In fibroelastic dysplasia, there is a decrease in connective tissue which is deficient in collagen, elastin, and proteoglycans.^36^ Although the leaflets present as thin and smooth and the chordae slightly elongated, thickened lesions have been noticed in the leaflet segments exposed to MR.^8,36^ Thus, isolated regions of the leaflets are pathologically involved.^35,36^ This leads to the hypothesis that the thickened lesions are secondary to MR.^8^

*Hypertrophic cardiomyopathy*

There is an ongoing discussion regarding the pattern of geometrical changes of the MV in HCM.

Thickening and elongation of the mitral leaflets are often encountered in HCM patients. Historically, abnormalities in HCM were thought to be limited to SAM. Recently it has been shown that this anomaly is not unique to HCM.^53^ SAM is caused by elongation of the anterior and/or posterior leaflet(s) predominantly along with anterior displacement of the papillary muscles that, with each contraction, come in contact with the septum further aggravating LV obstruction and conferring undue mobility to the mitral apparatus.^5^ It has been shown that LVH is a contributing but not necessary factor for left ventricular outflow tract obstruction (LVOTO). While the exact cause of the increase in size of the mitral leaflets is unknown, Patel et al. has shown that patients with infraclinical or with very little LVH can present LVOTO strictly in a phenotypic variant of HCM that affects anterior mitral leaflet length, attachment of the chordae tendineae and bifid papillary muscle mobility.^54^His work is supported by older papers that suggest a hemodinamically independent cause of mitral valve remodeling.^55-57^

This is in contrast to the belief that changes to the mitral apparatus are strictly a consequence of ventricular remodeling and ventricular mass.^58,59^

From this data Albert A. Hagège et al. has grouped the current available research into hypotheses.

*A. The Genetic Hypothesis*

A posterior leaflet elongation of >14mm was observed in carriers of a morbid HCM mutation, but without hypertrophy or obstruction.^60,61^ The presence of an enlarged leaflet in patients with HCM without hypertrophy or LVOTO suggests that changes in the geometry of the valve are not due to leaflet stress or ventricular remodeling, but rather depends solely on a genetic defect (a sarcomere gene mutation).

As mentioned above, MYBPC3 mutations are one of the causes of HCM. A study performed by Judge et al. shows that MYBPC3 knock-out mice with ecocardiographically diagnosed LVH have long and thick MV leaflets compared to controls. This abnormality occurs in the absence of LVOTO or SAM.^62^

*B. The Adaptive Hypothesis*

This is the theory that postulates that mechanical stress applied to the leaflets causes leaflet lengthening.^58,59^ A study performed on animals suggests that these changes might arise through reactivation of developmental embryonic pathways through endothelial-mesenchymal trans-differentiation (EMT). Cells synthesize α-smooth muscle actin in the atrial endothelium increasing collagen deposition. These changes are seen in the papillary muscles and chordae tendineae.^63-65^ As Hagège et al. concurs, this does not explain why leaflet enlargement occurs in the absence of SAM.

*C. The Concurrent Disease Hypothesis:*

The possibility of an independent MV disease that occurs with HCM has been brought up, namely the association between MVP and HCM. This theory has gained little traction due to the extensive studies and excellent diagnostic methods of MVP.^55^

*D. The Developmental hypothesis*

This hypothesis is developed from the feature of adult valves of being susceptible to embryonic signals that were active during fetal valvulogenesis. During myxomatous degeneration EMT is gradually reactivated. A surgical model shows similar changes occurring when MR is due to leaflet stretch.^63^

An explanation of the wide variance of phenotypic appearances of HCM has been postulated. Epicardial-derived cells (EPDCs) are normally found on the surface of the heart. Physiologically they differentiate into fibroblasts as they migrate into the ventricular and atrial walls and into cardiomyocytes as they migrate into the interventricular septum.^66^ It has been proposed that periostin is the stimulus which dictates the differentiation of EPDCs. When a sarcomeric gene mutation is present, EPDCs that have differentiated into cardiomyocytes become fibroblasts under the stimulus of periostin.^67^

Periostin is normally found in tissues derived from the mesenchyme, most commonly in osteoblasts, periosteum and the periodontal ligament. It is also involved in cancer development and the mesenchymal development of the heart.^66^ It directly affects restructuring of the ECM inducing remodeling and mediating response to tissue damage.^68^

Several other animal studies have shown increased levels of periostin in HCM mice and it has been consequently postulated that it may be implicated in valvular degeneration.^69,70^

**Figure 1:**
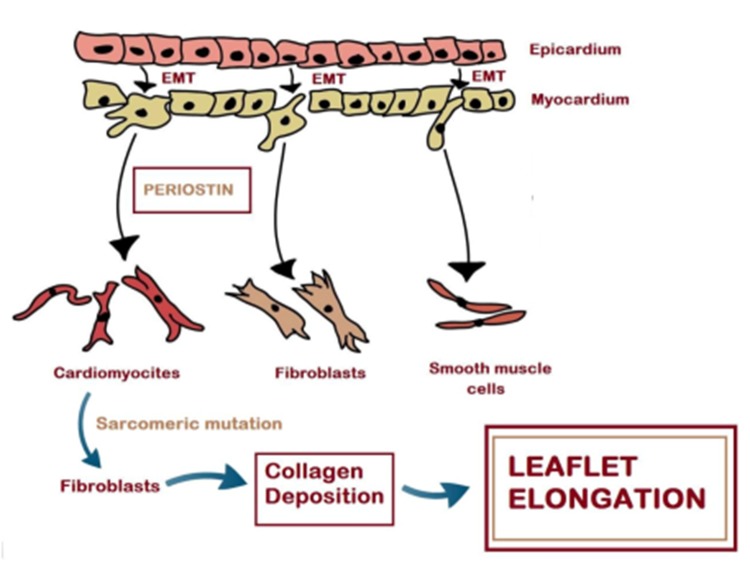
A proposed mechanism of leaflet elongation Through the process of EMT EPDCs migrate into the myocardium. Pathologically, cardiomyocytes in the interventricular septum (IVS) that have been formed from the EPDC line, transform under the stimulus of periostin into fibroblasts. Collagen deposition begins, which in turn explains the elongated leaflets commonly found in HCM patients.

New studies also suggest that in mice with HCM, genes that encode mutated contractile proteins cause EPDCs derived cardiomyocytes to become fibroblasts. This process occurs under an elevated level of periostin which may prove that periostin is responsible for collagen deposition on the MV, which may ultimately result in elongated leaflets (**Figure 1**).^71^

Theories abound, but a common mechanism that unites all theories is suspected.

## 5. Diagnosis

### 5.1 Mitral valve prolapse

MVP is the most common mitral abnormality of non-ischemic cause leading to severe surgery-requiring mitral regurgitation.^11,72^ It is defined by echocardiography as mono- or bileaflet (or limited only to scallops) systolic atrial displacement of at least 2 mm over the long-axis annular plane, that can be accompanied or not by leaflet thickening.^35,73,74^ The disease is characterised by typical fibromyxomatous changes of the leaflet tissue and possibly chordal abnormalities such as lengthening.^37,73^ According to the Framingham Heart Study, MVP affects approximately one in 40 individuals, irrespective of sex and age.^75^

Barlow's disease and fibroelastic dysplasia are the two different pathologies most commonly associated with MVP. Early presentation with multiscallop prolapse, diffuse thickening of the leaflets, lengthened chordae and severe annular enlargement is characteristic of Barlow's disease. Fibroelastic dysplasia, unlike Barlow's disease, presents later in life, involves isolated regions of the valve, rarely produces severe annular enlargement and frequently associates rupture of chordae.^35,36^

#### 5.1.1 Clinical evaluation

There is no specific symptomatology that characterises MVP. However, a constellation of symptoms (atypical chest pain, exertional dyspnea, palpitations, syncope, and anxiety) and clinical findings (leaner build, low blood pressure and electrocardiographic repolarization abnormalities) has been associated with this pathology under the name of MVP syndrome.^75,76^ The early misconception that this syndrome frequently occurs simultaneously with MVP was not confirmed by case-control studies.^35,74^

Symptoms occur when the compensatory ventricular dilation is overwhelmed and they indicate severe MR and LV dysfunction. Nonetheless, if the ventricular compensation of the severe MR is appropriate, the patient may remain asymptomatic for many years. Exertional dyspnea and exercise intolerance follow exhaustion of compensatory mechanisms and irreversible LV dysfunction occurs, as well as chordal rupture and sudden onset of AF.^35,72^

The importance of diagnosing MR as well as establishing its severity, lays in the excellent chance of improved quality adjusted life years for patients, provided they undergo intervention before symptomatology onset or irreversible ventricular damage.^72^ As far as the progression of the asymptomatic MVP to severe MR is concerned, there is evidence showing that 25% of the asymptomatic patients tend to develop a significant clinical form over the course of 3 to 16 years.^77^

When asymptomatic, this disease is usually discovered incidentally during clinical examination or during an echocardiogram.^74^MVP is more likely to be found in people having thoracic skeletal abnormalities and a leaner build.^76,78^

MVP is usually suspected following auscultatory findings, physical examination being a diagnostic standard.^75,79^ Classically, a dynamic, mid-to-late systolic click commonly associated with a late systolic murmur has been described.^74^ However, a late systolic murmur is more frequent than a mid-to-late systolic click.^79^

The click-dynamics may be perceived with specific maneuvers: an earlier click is produced by a reduced end-diastolic volume (e.g. Valsalva, standing), whereas opposite maneuvers generate a click shift into late systole.^74,80^

With chordal rupture and consequent leaflet flail, the murmur becomes holosystolic. A flailing posterior leaflet will generate a murmur radiating anteriorly which may mimic aortic stenosis.^35^

False positive auscultatory findings – non-prolapse related systolic clicks may occur. There are multiple causes for these findings such as bicuspid aortic stenosis, atrial myxoma and pericarditis. Nevertheless, echocardiographically documented prolapse may exist without auscultatory click or murmur.^74,80^ Thus, although a thorough physical examination is highly sensitive for echocardiographically documented MVP, its specificity is limited.^80^

#### 5.1.2 The role of echocardiography in MVP diagnosis

The primary diagnosis mean of MVP is echocardiography.^73,81^ Cardiac ultrasound provides information regarding valve morphology, severity of regurgitation, global and regional ventricular function and allows correlations between valve structure, biomechanics and haemodynamics.^5,73^ The standard investigations for patient assessment with MVP are two-dimensional (2D) and Doppler echocardiography.^82,83^ Newly implemented techniques of three-dimensional (3D) echocardiography, transthoracic echocardiography (TTE) and transesophageal echocardiography (TEE), permit visualisation of the MV from the so-called ‘surgical view’ thus enabling a better evaluation of the MV apparatus for pre-operative assessment.^73,81^

*Transthoracic echocardiography*

MVP is echocardiographically defined as valve leaflet displacement over the parasternal or apical long-axis annular plane during systole of more than 2 mm.^5,73^ Additionally, leaflet displacement in relation to each other (situation caused by chordal rupture or elongation, as well as enlargement of the mitral annulus) leads to malcoaptation, augmenting the severity of the regurgitation.^5^ Taking into account the saddle shape of the mitral annulus, distinct terms are used to describe leaflet displacement, while asserting prolapse magnitude.^1,5,84^ Thus, *billowing *refers to moderate degree prolapse when leaflet tips remain in the ventricle, (although leaflet bodies bulge into the atrium), while leaflet *flail *denotes severe prolapse when leaflet tip coaptation fails and both bodies and tips are displaced into the atrium.^1,5^

Valve characteristics that are evaluated by echocardiography in MVP are leaflet thickness and length, mitral annulus diameter and calcification, as well as the severity of MR.^5,72^ Other anatomical abnormalities that should be evaluated are the LV dimensions and function by means of measuring the ejection fraction and end-systolic and end-diastolic diameters and volumes.^72^

Generally, leaflets are elongated and thickened in MVP^5,51^ and it has been proven echocardiographically via parasternal long-axis view in diastole that mid-leaflet thickness is increased in 40% of patients with severe forms of this disease.^5,85^

**Figure 2:**
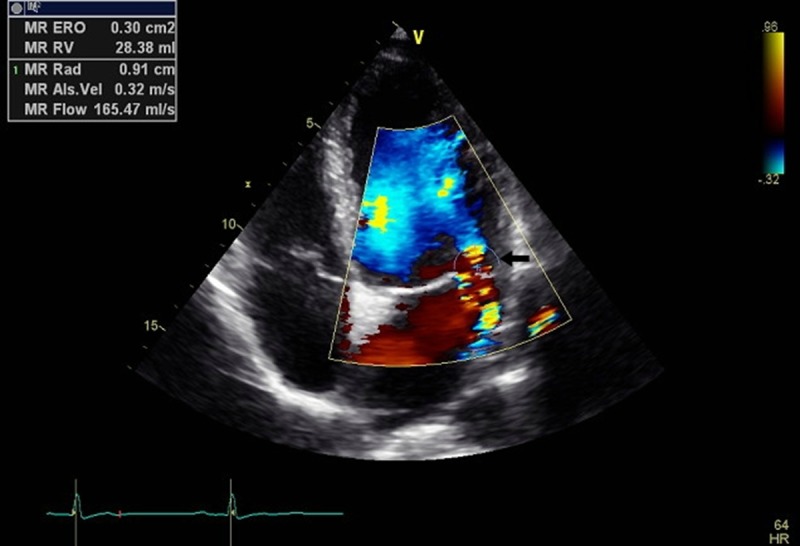
Transthoracic echocardiography showing moderate regurgitation EROA 30 mm^2^, Rvol 28 ml per beat; parameters measured with the help of PISA- standard quantitative assessment of MR

As mentioned, leaflet malcoaptation leads to MR. Establishing the severity of MR is highly important, as an intervention could be considered even in asymptomatic patients with severe MR in the presence of LV dysfunction.^82,83^ Doppler quantitative methods are more useful for grading the severity of MR^1,83^ compared with jet-based assessment which has major limitations. Proximal isovelocity surface area (PISA) method has become the standard quantitative assessment of MR.^1,86^ PISA allows measurement of effective regurgitant orifice area (EROA), regurgitant volume (RVol) and regurgitant fraction (RF).^1,81,83^ Regurgitation is considered severe in the setting of MVP when EROA is at least 40 mm^2^, RVol is at least 60 ml per beat and RF is at least 50%^1,81^ (**Figure 2**).

LV dimensions can also be performed by means of TTE. Volume overload is indicated by end-diastolic and end-systolic LV diameters and volumes, while ventricular function is assessed by end-systolic LV dimensions and LV ejection fraction.^1^ Ventricular dysfunction is diagnosed in the setting of severe MR due to MVP when the ejection fraction is less than 60% and the end-systolic diameter is higher than 45 mm.^83^

*The role of transesophageal echocardiography*

**Figure 3:**
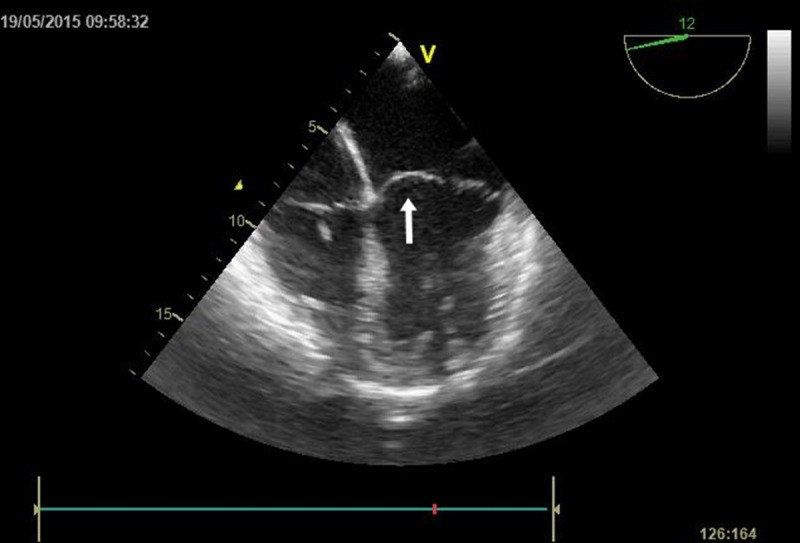
Transesophageal echocardiographic preoperative assessment of MVP showing prolapsed mitral leaflets (arrow) over the annular plane of at least 2 mm.

MVP imaging obtained via TEE is of higher quality compared to TTE, as it provides better assessment of the etiology and quantification of the severity of MR.^35,81,87^ It is preferred when TTE imaging is of poor quality or when complex, calcified or endocarditic lesions are being assessed.^1^ This investigation is mainly used on inpatients and during interventions to evaluate lesions and to monitor surgical results^88^ (**Figure 3**).

*The emerging role of three-dimensional echocardiography*

2D echocardiographical evaluation is complemented and its results are enhanced by 3D echocardiography, as the latter allows assessment of structures that might be problematic when using 2D echocardiography, such as the mitral annulus.^1,81^

3D TTE provides accurate data regarding anatomic localisation of prolapsing scallops, allowing better spatial localisation of commissures and quantification of MR severity.^81,89^ 3D multiplanar reconstruction (MPR) is a revolutionary echocardiography technique with an improved accuracy of MVP description compared to 2D investigations and the benefit of combining these two methods (3DMPR and 2DTTE) as part of the pre-operative investigations is increased.^11,89^

**Figure 4:**
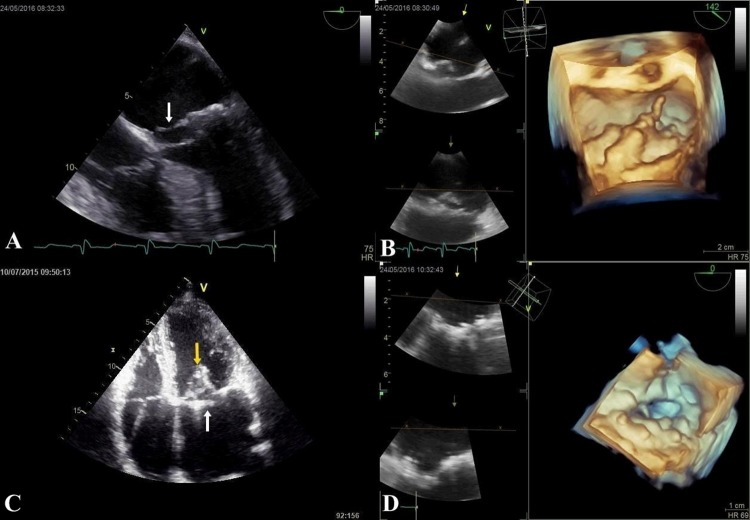
2D and 3D assessment of patient with MVP **A.** 2DTEE pre-operative imaging showing flail posterior leaflet (arrow); **B.** 3DTEE pre-operative imaging showing flail posterior leaflet( P2); **C.** 2D TTE apical four chamber view - post-operative imaging showing perfect coaptation (yellow arrow) and lack of leaflet prolapse (white arrow); **D.** 3DTEE post-operative imaging showing perfect coaptation and lack of leaflet prolapse.

3D TEE is superior to and has a distinct advantage over 2D TEE because imaging of the MV apparatus is more detailed and allows a more accurate localisation of prolapsing scallops^81,87,90^ (**Figure 4**). It has been shown that 3DTEE is comparable to cardiac magnetic resonance (CMR) regarding feasibility and accuracy of diagnosis.^81,91^

3D echocardiographic assessment of patients with MVP is now recommended for routine examination and its potential benefit is highlighted by current guidelines and studies.^11,81,83,89^

#### 5.1.3. The role of Cardiac Magnetic Resonance in MVP assessment

The main application of CMR insofar as MVP is concerned, is strictly for pre-surgical patient assessment.

Assessment of MR requires quantification of: grade of MR in order to determine the need for surgical intervention, the mechanism through which MR has appeared (leaflet prolapse with scallops), LV volumes for surgical risk assessment and myocardial viability in ischemic MR.^92^

In two studies comparing TEE and CMR ^(92,93)^ the two investigations were almost equally effective in diagnosing prolapsed or flailing leaflets with TEE actually proving slightly more effective in this regard.^93,94^

Although semi-invasive and operator dependent, TEE is excellent at accurately preparing patients for surgery and monitoring patient’s surgical response. CMR for MVP may be particularly useful in calculating LV dimensions in order to determine LV function and myocardial viability (it may also be used to measure the degree of fibrosis through late gadolinium enhancement (LGE) - gradual accumulation of a gadolinium based contrast agent followed by its delayed wash-out). Cost-effective analyses for this matter have yet to be performed.^94^

### 5.2 Mitral valve in HCM

HCM is an inherited autosomal-dominant myocardial disease caused by mutations in various genes encoding proteins of the cardiac sarcomere, which is characterized by significant LVH unexplained by either chronic pressure overload or infiltrative diseases.^95^

Due to an enhanced rate of recognition by means of echocardiography, as well as its autosomal dominant transmission, the prevalence of HCM in the general population is higher than it was previously estimated, with one in every 200 adults (0.5 percent).^96^

#### 5.2.1. Clinical evaluation and Electrocardiography changes in HCM

Symptoms can vary widely from individual to individual, comprising fatigue, dyspnea on exertion, anginal or atypical chest pain, palpitations and even syncope.^97^ It has been shown that symptoms may be exacerbated by the decrease in cardiac preload due to splanchnic vasodilatation postprandially.^98^

Patients with HCM who associate diastolic dysfunction are at an increased risk for supraventricular and ventricular arrhythmias, notably AF and even sudden cardiac death (SCD), especially in athletes.^99^ The prevalence of AF in the setting of HCM increases with age.^100^

Patients with HCM may associate LVOTO caused by the hypertrophied IVS.^97^

Notably, the presence of LVOTO worsens the prognosis by deteriorating left ventricular performance and by favoring the progression to more severe symptoms as well as raising chances of heart failure and death.^101^ Moreover, the SAM of the MV contributes to the development of MR and to the severity of the LVOTO, which represents a frequent cause of disabling symptoms in HCM.^102^

In the setting of LVOTO, physical examination of the patient may typically reveal a suggestive systolic murmur. On auscultation, LVOTO can be emphasized by a harsh crescendo-decrescendo systolic murmur at the apex that may radiate to the axilla and base.^97^

When these findings do not occur at rest, provocative maneuvers such as Valsalva maneuver or amyl nitrite inhalation reduce preload and may be used to augment the intensity of the murmur.^54^

Conversely, inspiration or hand grip increase afterload and therefore diminish the intensity of the murmur. For this reason, dynamic auscultation is crucial for the diagnosis of HCM.^103^

Physical signs of MR associated to HCM may include cardiomegaly and a laterally displaced apical impulse. It is essential to seek for a loud holosystolic murmur best heard at the apex which may radiate into the axilla.^1^

*Electrocardiography*

A routine electrocardiogram (ECG) is a sensitive yet not specific method to diagnose HCM.^97^ Signs of LVH associated with atrial enlargement (P wave abnormalities) due to persistent MR are highly suggestive for HCM. LVH results in increased precordial voltages and repolarization changes (horizontal or downsloping ST segment and T wave inversions). In the apical form of HCM, “giant negative T waves” in precordial leads may be observed in leads V2-V4. The axis may be deviated leftward.^1^

Deep, narrow Q waves in lateral (I, aVL, V5-V6) and inferior (II, III, aVF) leads indicate asymmetrical septal hypertrophy.

Furthermore, dysrhythmias such as supraventricular tachycardia may occur and even trigger malignant arrhythmias, eventually leading to SCD. AF is commonly seen in patients with advanced MR.^1^ Features of Wolff Parkinson White syndrome (short PR, delta wave) are frequently associated with HCM.^104^

#### 5.2.2 The role of echocardiography in HCM diagnosis

Further investigations using noninvasive cardiac imaging are required in order to establish the diagnosis of HCM and evaluate the severity of the pathological changes.

Performing an echocardiography on all patients suspected of HCM has the purpose of identifying the presence of cardiac morphology abnormalities such as LVH as well as appreciating the extent of systolic and diastolic dysfunction and diagnose the presence of MR.^100,105^

Comprehensive TTE is the initial imaging technique which demonstrates the pathological hallmarks of HCM: LVH, LVOTO and SAM of the MV.^106^

*Tranthoracic echocardiography*

2D echocardiography provides useful data concerning the distribution and extent of LVH, especially during diastole in parasternal short-axis plane. Alternative imaging windows are also used to confirm measurements.^97^

The criterion of a wall thickness exceeding 13 mm is used to diagnose HCM.^105^

The most commonly identified phenotypic variant of HCM is asymmetric hypertrophy, preferably localized within the basal segment of the anterior septum. This type is associated with LVOTO in 20-30% of cases^107,108^ and usually the LV ejection fraction is preserved. Evidence suggests that wall hypocontractility may develop before hypertrophy becomes evident.^109^

SAM of the MV consists in the anteriorly deviated movement of the mitral leaflets towards the ventricular septum during systole.^110^

In patients with HCM, flow through the narrowed LVOT is characterized by elevated velocities and reduced pressure of blood ejection (Venturi effect).^111^ The resultant pressure gradient leads to SAM.

In addition, structural abnormalities appear to be responsible for SAM of the MV.^1,102^ As evidence shows, SAM has been observed independently from HCM^112^ and precedes the increase of LVOT flow gradients.^113^

SAM may occur in a wide range of patterns, depending on whether one or both mitral leaflets are involved and the place of chordal attachment (usually to the distal parts of the leaflets).^114^

SAM of the MV may result in dynamic LVOTO due to the impingement of the MV leaflets upon the hypertrophied IVS. The severity of obstruction in the LVOT is highly influenced by SAM, as the longer the mitral leaflets are dragged anteriorly, the narrower the LVOT becomes and the flow velocity increases further.^102,115^

**Figure 5:**
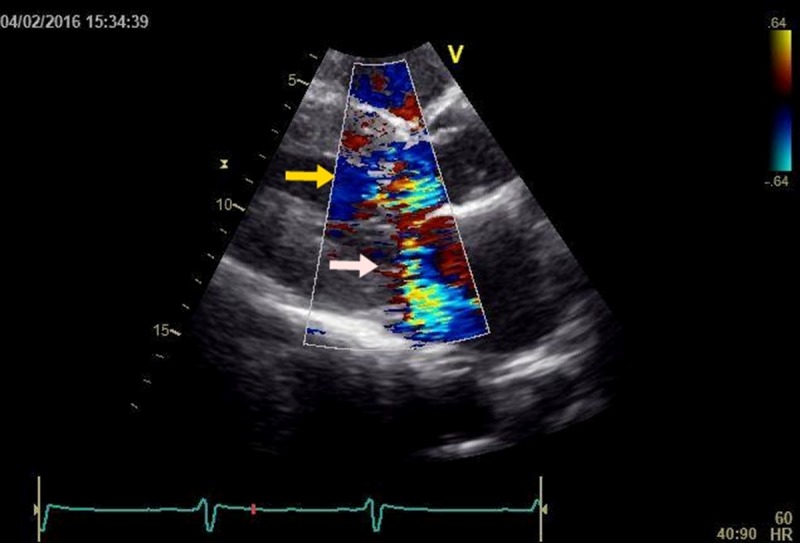
Transthoracic color Doppler assessment in the parasternal long axis view in HCM illustrating turbulent flow in the LVOT (yellow arrow) and a MR jet oriented posteriorly (white arrow)

LV outflow gradients are measured using continuous-wave Doppler imaging primarily in the apical long-axis view^3^. Increased velocities from the LVOT to the aortic valve plane due to SAM are marked by the turbulent appearance of the color Doppler flow^115^ (**Figure 5**).

Provocation tests using amyl nitrite, exercise or the Valsalva maneuver may be applied either for an enhanced diagnosis or for evaluating the response to pharmacologic agents.^97,106^

As leaflets are pulled into the LVOT during SAM of the MV, leaflet apposition becomes asymmetric and incomplete, resulting in a MR jet which is directed posteriorly.^106,110^

When the LVOT jet is present concomitantly with a MR jet, distinguishing signals from the two is challenging and the efficacy of a single flow assessment may be altered^116^ In terms of timing, LVOTO typically has a late systolic peak and a “dagger shaped” continuous-wave profile.

Conversely, MR ejection period lasts longer and usually reaches outstanding peak velocities of more than 6 m/s during mid-systole.^106^

The MR jet flow is easier to differentiate from LVOT jet flow when the Doppler beam is progressively oriented from the LVOT flow towards the MR flow. Furthermore, the two flows can be displayed separately when sweeping constantly from one flow to another.^117^

Evidence shows that leaflet length and mobility are important determinants of the severity of MR.^118^ For instance, when the posterior leaflet is not mobile or long enough to follow the anterior leaflet, malcoaptation occurs and an interleaflet gap results.^102^ Chordal and papillary muscle displacement also interferes with leaflet coaptation, as they impose the limits of mobility allowed.^119^

2D imaging gives information upon leaflet morphology and coaptation efficiency.

Continuous-wave Doppler evaluates velocities from the LV apex to the MV plane in an attempt to determine the peak flow of MR.^106^

A comprehensive examination is done in both parasternal and apical views from the medial to the lateral margins of the MV.^100^ Papillary muscle insertion abnormalities are easier to identify when using off-axis views with 2D echocardiography.^106^

*Transesopahgeal echocardiography*

TEE studies are mostly used for thorough periprocedural evaluation for planning purposes, although they are also useful when transthoracic views are not accurate enough.

While TTE shows elongated leaflets in patients coming to surgery^102,120^, TEE may also identify any additional concurrent organic cause of obstruction such as a subaortic membrane associated with dynamic obstruction.^105^

*Three-dimensional echocardiography*

By means of real-time 3D echocardiography the following morphological changes were identified to be responsible for SAM: leaflet elongation^121^ and thickening as a consequence of repetitive septal contact and hypertrophied, anteriorly displaced papillary muscles.^1,117^

#### 5.2.3. The role of Cardiac Magnetic Resonance in HCM assessment

**Figure 6:**
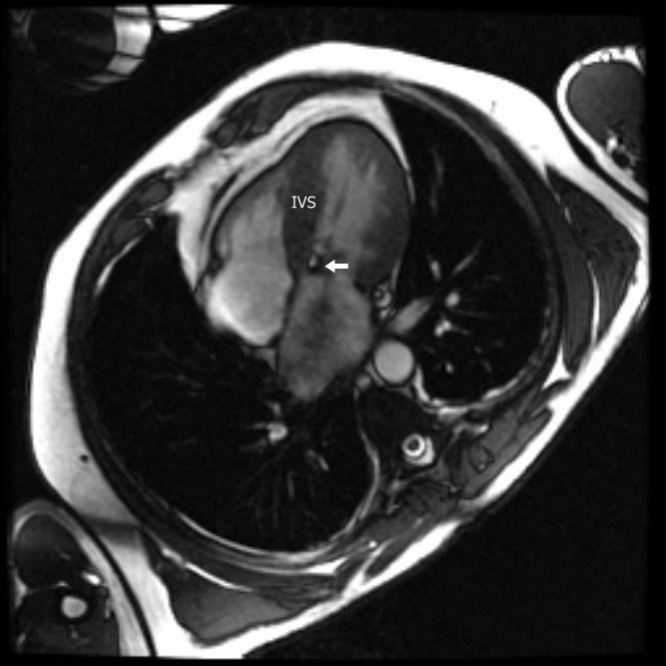
Four-chamber cardiac magnetic resonance image demonstrates marked hypertrophy of the IVS in a patient with HCM The arrow indicates the SAM of the MV.

As mentioned above, diagnosis of HCM traditionally relies upon clinical assessment and TTE in order to identify LVH, SAM and LVOTO. Heterogeneous phenotypic expression of the disease and technical limitations of echocardiography further complicate the diagnosis. CMR is an effective non-invasive imaging technique that can accurately measure LV mass^122,123^, degree of diastolic function (degree of LVOTO, presence of SAM^123^ (**Figure 6**) and possibly even the risk of SCD^124^) and it may also be used to distinguish between phenotypic variations of HCM (apical, symmetrical and asymmetrical HCM).^123^ CMR may also be used for monitoring post-surgical and post-pharmacologic treatment responses.^123-125^.

Particular cases of patients with non-diagnostic clinical and TTE investigations, but with positive HCM on CMR (with wall thicknesses up to 28 mm) have been reported^126^.

Insofar as technique is concerned, CMR benefits from the feature of various enhancement patterns in afflicted myocardium, namely LGE. The technique is based on gradual accumulation of a gadolinium based contrast agent followed by its delayed wash-out. LGE appears in a number of diseases that involve the myocardium, myocarditis, various cardiomyopathies and myocardial infarction being the most common ones.^107,124^

Non-invasive injection of the contrast agent helps describe tissue characteristics and may identify interstitial and replacement fibrosis typical for HCM. (LGE appears in 80% of patients with HCM, most commonly in the middle third of the left ventricular wall).^126,127^

Perhaps the most recent and important of CMR’s uses is the early detection of negative phenotypes, so called pre-clinical HCM. CMR studies by Maron et. al and Germans et al. have shown structural changes in the LV wall, in the form of *LV crypts*. Asymptomatic HCM patients were found to have crypts of up to 12 mm in the postero-basal ventricular septum which was undergoing hypertrophy. The significance of LV crypts remains to be determined.^64,65^

## 6. Treatment

### 6.1 Mitral Valve Prolapse Management

#### 6.1.1 Pharmacological treatment

Asymptomatic patients with minimal disease need an initial risk stratification by means of echocardiographic evaluation. If clinically significant MR is absent and thin leaflets are observed, the patient is encouraged to pursue a normal lifestyle, with clinical examinations and echocardiographic evaluation at every 3-5 years.^128^

In symptomatic MR and/or left ventricular dysfunction associated to MVP, afterload-reducing agents (nitrates, diuretics and other antihypertensive drugs) are used for maintaining cardiac output. Caffeine, alcohol and cigarettes should be avoided.

In AF, maintenance of a normal ventricular response is ensured by administering beta-blockers and calcium channel blockers. Anticoagulants, such as anti-vitamin K drugs, are also recommended especially in patients with risk factors but also for the ones post MV replacement surgery.^128,129^

The use of prophylactic antibiotics prior to periodontal and surgical procedures, although highly recommended in the past, has been limited to selected cases in recent years, according to the American Heart Association (AHA) guidelines.^83^

Inotropic agents should be used in chronic severely symptomatic MR.^129^

ESC/EACTS guidelines suggest diminishing filling pressures in acute MR (with or without hypotension) with nitrates and diuretics, as well as afterload and regurgitant fraction reduction by means of nitroprusside therapy or an intra-aortic balloon pump.

In the case of hemodynamic shock, inotropic agents and an intra-aortic balloon pump should be used.^82^

#### 6.1.2. Surgical treatment

Although pharmacological therapy might relieve symptoms in severe MR or LV dysfunction (ejection fraction <60% or end-systolic diameter >45 mm), it cannot alter the natural development of the disease, surgery remaining the ultimate treatment^72^. The purpose of surgical correction in MVP is to restore MV competence.

*Mitral valve replacement*

An option for surgical correction is MV replacement. It can be performed using either a mechanical, or a biologic prosthesis. Although this method is considered to have the highest long-term efficacy, it has several drawbacks. Mechanical valves pose a risk of thromboembolism, thus needing lifelong anticoagulation therapy, while bioprosthetic valves rapidly deteriorate. Additionally, there is a high susceptibility of prosthetic-valve endocarditis or chordal cleavage during surgery.^130^

*Mitral valve plasty*

Surgical repairs are the preferred treatment for MVP. Although being associated with a higher post-operative recurrence of MR (in comparison with valve replacement), mitral plasties have a lower peri-operative mortality.^83,131^ MVP is usually repaired, either by resecting the flail and prolapsing leaflet scallop or by reconstructive techniques with artificial polytetrafluoroethylene (PTFE) chords. Annular dilation is often associated with MR and is corrected with a complete or partial annuloplasty ring. Posterior leaflet prolapse is more common, leading to severe MR. However, it has a higher success rate of durable repair compared with anterior leaflet prolapse or severe bileaflet prolapse. Ultimately, the most important indicator of long-term life expectancy in mitral repair remains the expertise of the surgeon.

Despite median sternotomy being the standard surgical approach, minimally invasive techniques are increasingly used nowadays (representing 21% of surgical procedures), while the robotic approach is also gaining popularity. Both methods include partial sternotomies and restricted or mini-right thoracotomies.^72^

*Transcatheter mitral valve repair*

A cutting-edge method in MVP treatment is the transcatheter MV repair. The technique that mimics the surgical procedure proposed by Alfieri et al. offers an alternative to surgery for patients with symptomatic severe MR and high surgical risk. MitraClip therapy consists in edge-to-edge leaflet repair by clipping together the free edges of leaflets at their mid-portion. The Endovascular Valve Edge-to-Edge Repair Study (EVEREST II) compared MitraClip to surgery, showing that percutaneous repair of the mitral valve was less effective in reducing MR. However, this type of transcatheter repair was associated with similar improvement in clinical outcomes and with superior safety.^72,129^

*Papillary muscle shortening*

Papillary muscle shortening is another technique for MVP treatment used in the cases of abnormal muscle anatomy. There are two approaches for this technique: one by cutting a muscle section. An alternative method involves placing and tying a suture through the top of each muscle end and then passing it through the base, shortening them.^132^ Papillary muscle repositioning was also proposed as a reliable and safe technique in the repair of anterior leaflet prolapse, with excellent clinical and echocardiographic long-term results.^133^

*Replacement of chordae tendineae*

While mitral scallop “resection” methods have provided excellent long-term results, there is increasing evidence that supports “non-resection” techniques, with a “respect rather than resect” philosophy.^134^ Options to manage elongated chordae in MVP include natural chordal transfers (rarely used nowadays) or PTFE neochord replacements.^132^ Potential advantages of the “non-resection” approach include: preserved leaflet mobility, larger coaptation surface, implantation of larger prosthetic annuloplasty ring, which provides lower gradients due to larger orifice area, and lack of changes in annular geometry.^134^

However, some improvements have been brought to the PTFE chordal replacement technique, as the classic procedure involves open-heart surgery.^135^ During the last 6 years, two transcatheter techniques of placing artificial chordae have been developed^72^ The correct length of the chordae is determined by real-time echocardiographic guidance while observing the improvement in MR (as opposed to the traditional method of approximation based on the length of the adjacent chordae and the result of the “saline test”).^132,135^

### 6.2 Management of mitral valve in HCM

#### 6.2.1 Pharmacological treatment

In asymptomatic patients, the main concern is instructing them regarding the management of their disease. Intense physical activities should be avoided, changing their lifestyle should also be encouraged. Treatment of risk factors is also of paramount importance. Pharmacological therapy is usually the first line of treatment in order to prevent complications and disease progression. On an empirical basis, beta blockers and calcium channel blockers are the elected medications. Invasive treatment techniques are not recommended for these patients.^105^

In symptomatic patients, the main concerns are treating symptoms, improving functional capacity and postponing the invasive treatment techniques. Symptoms such as angina or dyspnea should be treated using beta-blockers. For patients who present side effects or contraindications to beta-blocking drugs, verapamil or diltiazem therapy is recommended. AF should be managed using anticoagulation with vitamin K antagonists or direct thrombin inhibitors and anti-arrhythmic such as amiodarone, disopyramide or sotalol.^136^

#### 6.2.2. Invasive therapies

Patients with progressive disease (severe symptoms which interfere with everyday activity and quality of life, dynamic LVOT gradient greater than 50 mmHg or severe septal thickness) and who are refractory to medical therapy are suitable candidates for the following: septal myectomy with or without MV repair, alcohol septal ablation, chordae resection, papillary muscles surgery or even heart transplant.^137-142^

*Septal myectomy*

It is a surgical procedure aimed at reducing the muscle thickening by removing a small amount of the thickened septal wall to decrease LVOTO^140^ and improve the MR grade. A study showed that after septal myectomy, all of the patients experienced a significant reduction in MR degree: no MR in 80%, mild in 19%, moderate in 1%.^142^ The main complications were atrioventricular nodal block, ventricular septal defect and aortic regurgitation.^137^

This method is now associated with a less than 1% mortality risk^143^, including a decrease in the LVOT gradient from 78 mmHg to 11 mmHg and an improvement in 98% of the patients concerning the NYHA class (I, II).^144,145^

*Septal myectomy and mitral valve repair*

Whereas the surgical septal myectomy is the gold standard procedure in treating HCM patients, it does not directly address the involvement of the MV in the pathophysiology of HCM. A two-step procedure seems more efficient: septal myectomy, followed by MV repair with reconstruction.^138^

Excellent improvements have been observed, regarding the haemodynamics of the MV and clinical symptoms: NYHA class III and IV patients reached NYHA class I; the control echocardiography showed no MR, with a mean resting gradient of 15 mmHg (from a prior 73.5 mmHg).^138^ Other studies also support the dual method, as the data suggests a more satisfactory outcome compared to myectomy alone.^146^ Another study has also shown a significant clinical improvement in patients that underwent septal myectomy combined with MV repair, rather than in patients that underwent septal myectomy alone.^147^

*Septal myectomy and chordae resection*

Another surgical option could be septal myectomy combined with the resection of the MV anterior leaflet chordae. A long-term study analysed the efficiency of this technique (resection of the septal muscle and papillary muscles mobilization, followed by cutting the deteriorated chordae). The results showed improvements regarding the NYHA class: from class III-IV to asymptomatic and class I-II post-surgery. Also the LVOT gradient changed from 82 to 9 mmHg and the MR changed from grade 3 and 4 to lower grades.^140^

*Papillary muscles surgery*

Papillary muscle surgery is the surgical approach that should be considered regarding displacement of the base of the anterolateral papillary muscle or its abnormal muscular insertions into or near the A1 scallop. Studies have shown the possibility of a longitudinal excision to thin the abnormal papillary muscle which caused the obstruction.^102^ The surgical reorientation is performed to prevent ischemia, by suturing the fibrous portion of the papillary muscle in order to realign them as necessary and to reduce the hypermobility. The mean perioperative (114 mmHg) and predischarge (16 mmHg) LVOT gradients, show the effectiveness of this method. Moreover, the results have been compared to those of the patients who underwent isolated myectomy and myectomy plus MV repair/replacement, outlining the similarities regarding the outcome and concluding the surgical alternative for those who do not meet the septal thickness criterion for myectomy.^148^

*Alcohol septal ablation*

Alcohol septal ablation (ASA) is a percutaneous, minimally invasive procedure which induces localized myocardial infarction, resulting in the thinning of the IVS, therefore leading to the reduction in dynamic outflow obstruction. For this method to be applied, LVOT should have a gradient more than 50 mmHg.^149^ It is considered an alternative to septal myectomy when the latter is unsuitable. The reported survival rate at 5 years is 91.7%. However, the procedure should not be encouraged in patients with marked septal hypertrophy (>30 mm), who do not meet the age requirements (>21 years of age) and have concomitant heart diseases that need surgical correction (coronary artery bypass, ruptured chordae of the MV). Another important aspect is the risk of developing post-procedural AF or atrioventricular block.^139^

*Pacing and Implantable cardioverter defibrillators (ICDs)*

Permanent pacing and ICDs are suitable for patients who cannot undergo surgical treatment and are at risk of developing life-threatening arrhythmias.^105^ Despite the low mortality rate, complications are common, therefore the benefits and risks of this therapy should be carefully weighted.^150-153^

*Heart transplant*

HCM patients represent a small subset (<1%) of the whole patients undergoing heart transplant.^154^ Nonetheless, their survival rates after this procedure are comparable to those of patients transplanted for other cardiovascular diseases: the survival rates at 1, 5 and 10 years were 85%, 75% and respectively 61%.^141^

### 6.3 Clinical outcome

*Mitral Valve Prolapse***

The outcome of patients with MVP mainly depends on the chosen treatment.

Surgical treatment comprises of either repairing or replacing the MV.

In terms of replacement surgery, bio- and mechanical prostheses have similar mortality and follow-up rates. Bleeding risk evaluation offers varying results with some studies showing a more significant risk of bleeding for mechanical valves^155,156^ while others show comparable results. Bio-prostheses are more prone to reoperation. Therefore, the type of prosthesis cannot accurately predict the outcome of MVP patients who underwent valvular surgical replacement.^157^

MV repair carries a vastly lower mortality compared to replacement surgery both immediately following surgery and on long-term follow-up (it has been shown that patients undergoing MV replacement have a 58% higher 5 year mortality rate). This tendency is also valid for myxomatous valvulopathies that required surgical treatment, making valvular repair the elective treatment for patients with MVP.^158^

*Hypertrophic cardiomyopathy*

Patients under 65 years of age who underwent septal myectomy had better survival rates and lower severity of symptoms than patients who underwent ASA. ASA patients became completely asymptomatic in only about half of the cases.^159-162^ Over 65 years of age, however, ASA had a more significant decrease in gradient and symptoms than septal myectomy. Regarding mortality, certain studies have shown similar peri-procedural rates^162,163^, while others have shown a higher incidence of complications in the case of ASA.^164,165^ There is an ongoing debate which procedure should be favored, this being highly dependent on patient types and operative expertise.^166^

A study compared LVOT gradient, MR and SAM in patients who underwent septal myectomy and concomitant MV repair or MV replacement. Preoperative peak LVOT gradient was 63.7 mmHg with severe MR present in 88% of the cases and with SAM in 94%. Following surgery LVOT gradient decreased to 10 mmHg, 21% of patients, still presented chordal SAM and MR was absent or mild in 75% of cases and moderate in 21%. At late follow-up, LVOT decreased to 2.5 mmHg, SAM was no longer present in any of the patients and only 2 had MR and 84% of patients were in NYHA class I or II.^155^

### 6.4 Future perspectives

#### 6.4.1 Surgical novelties for MVP

Surgery is prone to remain the gold standard therapy in low-risk or intermediate-risk patients with MVP.^72^

The new trend nowadays is developing minimally invasive techniques.^72,135^ Supporting this concept, robotic surgery has come to the aid of MVP treatment. This technique allows increased operative dexterity and ambidexterity, higher degree of movement freedom (compared with the endoscopic approach), tremor-free movements, by-pass of the fulcrum effect (often manifested during endoscopic procedures) and use of 3D high definition imaging. Furthermore, patients directly benefit from the smaller incisions, shorter length of hospitalisation, sooner return to the pre-operative level of functional activity and decreased blood transfusion requirements. Some limitations in robotic MV surgery remain the increased operative times and lack of haptic feedback. Morbidity and mortality rates are similar to both classic cardiac surgery and minimally invasive procedures.^167^

Additionally, a novel patient-specific virtual valvular repair strategy was implemented, in order to decide pre-operative selection of the optimal repair techniques. Personalized computational modeling for MV repair, along with 3D echocardiographic guidance can provide higher outcome predictability and, ultimately, more effective medical care for the patient.^168^

In the pursuit of overcoming some of the MV replacement limitations, tissue engineering-based techniques have been approached. Among various scaffolds used in tissue engineering, the decellularized heart valve (DHV) has attracted much attention, as a result of its native structure and comparable haemodynamic characteristics. The clinical application of DHV remains under debate (due to insufficient recellularization, risk of immunogenicity and calcification).^169^

#### 6.4.2 Gene therapy for HCM

The applicability of gene therapy is limited to dominant monogenic muscle disorders such as HCM, mitigating the consequences of myofilament mutations. Limitations comprise the receptivity of muscle to gene transduction, gene delivery using vectors such as adeno-associated virus 9 (AAV9)^170^ and the attainment of a controlled expression of exogenous genes.^171^ Systemic administration of cardiac adeno-associated virus (AAV) and a cardiomyocyte-specific promoter specifically targets the heart.

In severe cases of inherited cardiomyopathy associating systolic heart failure with a heart transplant as an ultimate treatment option, gene therapy plays an important role. Commonly, homozygous and double heterozygous mutations in the sarcomeric genes predispose to rapidly progressive cardiomyopathies.^172^

Gene therapy may either remove mRNA mutations or introduce functional proteins in the place of mutant endogenous ones. Techniques directly targeting endogenous mutant sarcomeric mRNA or RNA are spliceosome-mediated RNA trans*-*splicing, exon skipping and RNA silencing.^173^

Spliceosome-mediated RNA trans*-*splicing (SMaRT) is an approach which creates a full-length repaired mRNA by splicing together a mutant pre-mRNA and a therapeutic pre*-*trans-splicing molecule, both transcripted independently (delivered by AAV).^174^

Different strategies use antisense oligonucleotides (AONs) to mediate the skipping of mutated exons. Their role is to restrict the binding of regulatory splicing proteins that mediate exons inclusion into the mature mRNA by concealing exonic splicing enhancer motifs.^175^ Despite the small internal deletion, the protein’s functionality is unaltered. AAV9 administration in myosin-binding protein C 3 – knock in mice (MYBPC3-KI mice) produced a stable functional protein which temporarily prevented the disease phenotype.^176^ However, reaching the appropriate level of intracellular oligonucleotide that entirely suppresses missense mutations remains a major challenge.^171^

Severe sarcomeric cardiomyopathies due to bi-allelic MYBPC3 mutations have been associated with fewer mutant proteins. The causal therapy in this particular case consists of replacing endogenous mutant myosin binding protein-C (cMyBP-C) with exogenous functional cMyBP-C. In a recent study performed on homozygous MYBPC3-KI mice, one systemic injection of AAV9 encoding cMyBP-C under the control of human cardiac troponin T promoter in neonatal mice has enabled a 34 week long prevention of the disease phenotype, namely LV hypertrophy.^177^

Cardiac troponin C (cTnC) is the target of genetic manipulation with the aim to reverse primary functional changes of the sarcomeres. Newly formed cTnC mutants are produced in order to behave as Ca^2+^ desensitizers owing to their intrinsic Ca^2+^-binding properties. Signaling of muscle contraction is impeded by compounds that bind to either the N-terminal regulatory domain or the C-terminal structural domain of cTnC. Consequently, the Ca^2+^-dependent interaction between cTnC and cardiac troponin I (cTnI) is prevented. Furthermore, cTnC is structurally homologous with calmodulin (CaM), making CaM-binding compounds potential modulators of Ca^2+^ sensitivity in the cardiac myofilament.^173^

Vertically transmitted HCM may be prevented when a couple undergoes assisted reproduction by in vitro fertilization. First,one cell is removed from each embryo and analysed for specific mutations. Subsequently, mutation free embryos are selected and transferred into the uterus.^178^

Conclusively, based on the fact that HCM does not significantly affect longevity, genetic engineering is not a priority nowadays. Gene therapy is mostly suitable for recessively inherited defects induced by mutations which partially or completely alter enzyme function.^179^

## 7. Conclusions

The mitral valve undergoes similar leaflet elongation and thickening changes, although through different mechanisms, in mitral valve prolapse and in hypertrophic cardiomyopathy. These mechanisms involve disruption of the homeostasis of the extracellular matrix in mitral valve prolapse, while in hypertrophic cardiomyopathy there are many theories, but a common mechanism is suspected. Both pathologies involving the mitral valve have genetic causes: in mitral valve prolapse, most, if not all changes, revolve around the activity of TGF-β, whereas the mutations responsible for the modifications in hypertrophic cardiomyopathy are in genes that mostly codify sarcomeric proteins.

The role of cardiac imaging is vital for diagnosing both pathologies and the consequent mitral regurgitation. Three-dimensional echocardiography has an emerging role in mitral valve prolapse diagnosis and is comparable to cardiac magnetic resonance imaging in the context of this disease. Also, three-dimensional echocardiography is routinely recommended and has a high potential benefit. Cardiac magnetic resonance imaging emerges in the diagnosis of hypertrophic cardiomyopathy through an accurate measurement of characteristic features and it could possibly even evaluate the risk of sudden cardiac death.

Surgery is the curative treatment for these diseases involving the mitral valve. Mitral valve repairs are suitable interventions for the couple of pathologies that induce mitral regurgitation. While mitral valve prolapse only requires mitral plasty, in hypertrophic cardiomyopathy septal myectomy and mitral valve repair are required. In addition, cutting edge techniques for treating mitral valve prolapse were developed, such as decellularized heart valve, robotic surgery and virtual valve repair simulation. Regarding future perspectives for hypertrophic cardiomyopathy, gene therapy is a promising field, directly targeting endogenous mutations in the sarcomere.

## Bullet Points

◊ **High levels of TGF-β are involved in the pathogenicity of mitral valve prolapse**

◊ **Sarcomeric protein mutations are mostly incriminated for the occurrence of hypertrophic cardiomyopathy**

◊ **An accurate description and a better understanding of the process of leaflet elongation will be essential in establishing new therapies for the treatment of both diseases**

◊ **Early diagnosis, grading and treatment of mitral regurgitation before symptomatology onset or irreversible ventricular damage lead to an excellent chance of improved quality adjusted life years**

◊ **3D transesophageal echocardiography is comparable to cardiac magnetic resonance regarding feasibility and accuracy of diagnosis in mitral valve prolapse**

◊ **Three-dimensional echocardiographic assessment of patients with mitral valve prolapse is now recommended for routine examination**

◊ **Multimodality imaging enhances the diagnosis accuracy in patients with hypertrophic cardiomyopathy and identifies the structural abnormalities in the mitral valve responsible for the presence of systolic anterior motion**

◊ **In patients with hypertrophic cardiomyopathy and dynamic left ventricular outflow tract obstruction, transesophageal echocardiography may recognize additional concurrent organic causes of obstruction such as a subaortic membrane**

◊ **Cardiac magnetic resonance imaging is an increasingly useful tool in diagnosing infra-clinical cases of hypertrophic cardiomyopathy**

◊ **Personalized computational modeling for mitral valve repair along with three-dimensional ecocardiographic guidance can provide a pre-operative selection of the optimal repair technique**

◊ **For severe cases of hypertrophic cardiomyopathy, a promising therapeutic alternative is gene therapy, using different strategies to remove mRNA mutations or to replace mutated endogenous proteins with functional ones, in this manner delaying phenotypic expression**
